# Poly[[aquadi-μ_3_-malonato-hexaphenyl­ditin(IV)] acetone solvate]

**DOI:** 10.1107/S1600536810018398

**Published:** 2010-05-22

**Authors:** Yip Foo Win, Siang Guan Teoh, M. R. Vikneswaran, Jia Hao Goh, Hoong-Kun Fun

**Affiliations:** a Department of Chemical Science, Faculty of Science, Universiti Tunku Abdul Rahman, 31900 Kampar, Perak, Malaysia; b School of Chemical Sciences, Universiti Sains Malaysia, 11800 USM, Penang, Malaysia; c X-ray Crystallography Unit, School of Physics, Universiti Sains Malaysia, 11800 USM, Penang, Malaysia

## Abstract

The asymmetric unit of the title polymeric complex, {[Sn_2_=(C_6_H_5_)_6_(C_3_H_2_O_4_)(H_2_O)]·C_3_H_6_O}_*n*_, comprises of two Sn cations, one malonate anion and a non-coordinating acetone solvent mol­ecule. Both crystallographically independent Sn cations are five-coordinated by two O and three C atoms in a distorted trigonal-bipyrimidal geometry. One of the Sn cations is bridged by the malonate units, affording polymeric chains which run along [001]. Weak intra­molecular C—H⋯π inter­actions stabilize the mol­ecular structure. In the crystal structure, adjacent chains are inter­connected by inter­molecular O—H⋯O and C—H⋯O hydrogen bonds into a three-dimensional supra­molecular structure. A weak inter­molecular C—H⋯π inter­action is also observed.

## Related literature

For general background to and applications of the title complex, see: Ng (1998 ▶); Ng & Kumar Das (1993 ▶); Ng *et al.* (1990 ▶); Samuel-Lewis *et al.* (1992 ▶). For a related bis­(triphenyl­tin) structure, see: Ng (1998 ▶). For the stability of the temperature controller used for the data collection, see: Cosier & Glazer (1986 ▶).
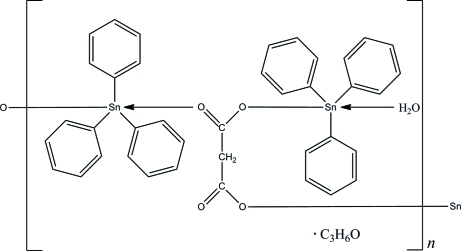


## Experimental

### 

#### Crystal data

[Sn_2_(C_6_H_5_)_6_(C_3_H_2_O_4_)(H_2_O)]·C_3_H_6_O
*M* *_r_* = 878.12
Tetragonal, 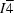

*a* = 23.604 (3) Å
*c* = 13.8458 (18) Å
*V* = 7714.2 (17) Å^3^
*Z* = 8
Mo *K*α radiation
μ = 1.34 mm^−1^
*T* = 100 K
0.22 × 0.13 × 0.04 mm

#### Data collection

Bruker APEXII DUO CCD area-detector diffractometer
Absorption correction: multi-scan (*SADABS*; Bruker, 2009 ▶) *T*_min_ = 0.754, *T*_max_ = 0.953
68157 measured reflections
8900 independent reflections
7984 reflections with *I* > 2σ(*I*)
*R*_int_ = 0.099

#### Refinement

*R*[*F* ^2^ > 2σ(*F* ^2^)] = 0.051
*wR*(*F* ^2^) = 0.103
*S* = 1.17
8900 reflections
421 parameters
3 restraints
H-atom parameters constrained
Δρ_max_ = 0.97 e Å^−3^
Δρ_min_ = −1.07 e Å^−3^
Absolute structure: Flack (1983 ▶); 4262 Friedel pairs
Flack parameter: 0.04 (3)

### 

Data collection: *APEX2* (Bruker, 2009 ▶); cell refinement: *SAINT* (Bruker, 2009 ▶); data reduction: *SAINT*; program(s) used to solve structure: *SHELXTL* (Sheldrick, 2008 ▶); program(s) used to refine structure: *SHELXTL*; molecular graphics: *SHELXTL*; software used to prepare material for publication: *SHELXTL* and *PLATON* (Spek, 2009 ▶).

## Supplementary Material

Crystal structure: contains datablocks global, I. DOI: 10.1107/S1600536810018398/ng2776sup1.cif

Structure factors: contains datablocks I. DOI: 10.1107/S1600536810018398/ng2776Isup2.hkl

Additional supplementary materials:  crystallographic information; 3D view; checkCIF report

## Figures and Tables

**Table 1:** Selected interatomic distances (Å)

Sn1⋯O1	2.333 (4)
Sn1⋯O3^i^	2.148 (4)
Sn1⋯C1	2.124 (7)
Sn1⋯C7	2.132 (6)
Sn1⋯C13	2.133 (7)
Sn2⋯O2	2.164 (4)
Sn2⋯O1*W*	2.325 (4)
Sn2⋯C19	2.137 (7)
Sn2⋯C25	2.139 (7)
Sn2⋯C31	2.119 (7)

Symmetry code: (i) 
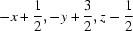
.

**Table 2:** Hydrogen-bond geometry (Å, °) *Cg*1 and *Cg*2 are the centroids of the C31–C36 and C7–C12 benzene rings, respectively.

*D*—H⋯*A*	*D*—H	H⋯*A*	*D*⋯*A*	*D*—H⋯*A*
O1*W*—H1*W*1⋯O4^ii^	0.86	1.90	2.663 (6)	148
C5—H5*A*⋯O5^iii^	0.93	2.59	3.38 (3)	144
C26—H26*A*⋯O4^ii^	0.93	2.50	3.356 (8)	154
C8—H8*A*⋯*Cg*1	0.93	2.83	3.701 (8)	157
C17—H17*A*⋯*Cg*2^iv^	0.93	2.79	3.571 (9)	142
C38—H38*B*⋯*Cg*2	0.97	2.97	3.613 (8)	125

Symmetry codes: (ii) 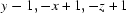; (iii) 
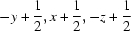
; (iv) 
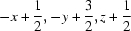
.

**Fig. 1.**
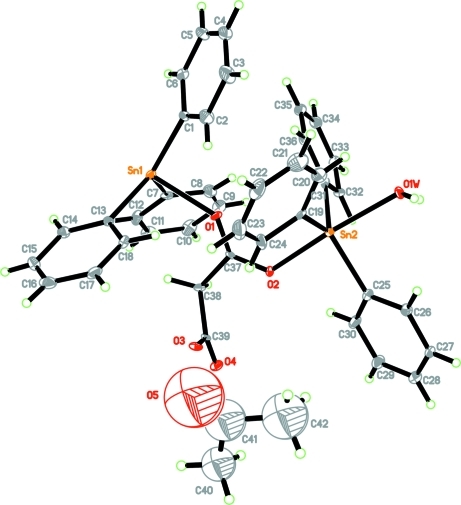
The asymmetric unit of the title polymeric complex, showing 30% probability displacement ellipsoids for non-H atoms and the atom-numbering scheme.

**Fig. 2.**
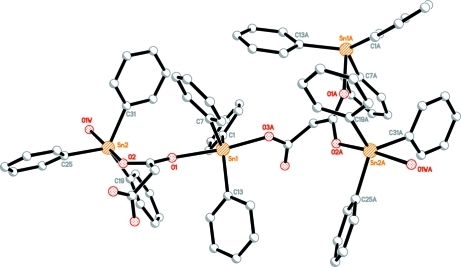
Part of the polymeric chain, showing the coordination environment of Sn cations. Non-coordinating acetone solvent molecule and H atoms have been omitted for clarity. Symmetry codes: (a) -x+1/2, -y+3/2, z-1/2 (b)-x+1/2, -y+3/2, z+1/2

**Fig. 3.**
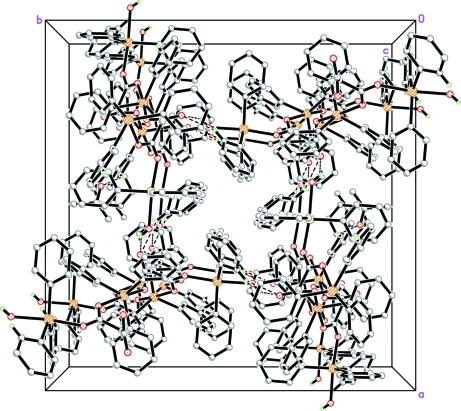
The crystal structure of the title polymeric complex, viewed along the c axis, showing the polymeric chains being linked into a three-dimensional supramolecular structure. H atoms not involved in intermolecular hydrogen bonds (dashed lines) have been omitted for clarity.

## supplementary crystallographic information

### Comment

The studies of organotin(IV) carboxylate derivative complexes of dicarboxylic acids have been documented since 1990s and various kind of bis(triorganostannyl) esters of substituted aliphatic dicarboxylic acids have been prepared (Ng, 1998; Ng & Kumar Das, 1993; Ng *et al.*, 1990; Samuel-Lewis *et al.*, 1992). Moreover, the crystal structure of bis[triphenyltin(IV)] succinate and its complexes have also been reported (Ng, 1998; Ng & Kumar Das, 1993). However, the crystal structure of bis[triphenyltin(IV)] derivative of malonic acid has not been reported. In this study, the structure of the title complex is similar to bis[triphenyltin(IV)] succinate. The exception is that the water molecule coordinates to the tin cation.

The asymmetric unit of the title polymeric complex comprises of two crystallographically independent Sn cations (Sn1 and Sn2) and a non-coordinating acetone solvent molecule (Fig. 1). Both Sn cations are five-coordinated by two O and three C atoms. The coordination geometries are distorted from the ideal trigonal bipyrimidal geometry, resulting in see-saw shaped geometries. The coordination environments are different for the two Sn cations (Fig. 2). The Sn1 cation is coordinated to three phenyl ligands and two carbonyl O atoms, forming one-dimensional polymeric chains along the [001] direction whereas the Sn2 cation is coordinated to three phenyl ligands, a water molecule and a carbonyl O atom. Further stabilization of the molecular structure is provided by the weak intramolecular C8—H8A···*Cg*1 and C38—H38B···*Cg*2 interactions (Table 2). The O—Sn1—O and O—Sn2—O angles are 174.03 (17) and 173.79 (18)°, respectively. Bond lengths of Sn—O and Sn—C are listed in Table 1. All bond lengths and angles are comparable to a closely related bis(triphenyltin) structure (Ng, 1998).

In the crystal structure (Fig. 3), adjacent polymeric chains are interconnected into a three-dimensional supramolecular structure by intermolecular O1W—H1W1···O4, C5—H5A···O5 and C26—H26A···O4 hydrogen bonds (Table 2). The crystal structure is further stabilized by weak intermolecular C17—H17A···*Cg*2 (Table 2) involving the centroid of the C7-C12 (*Cg*2) benzene ring.

### Experimental

The title complex was obtained by heating under reflux a 2:1 molar mixture of triphenyltin(IV) hydroxide (4 mmol, 1.47 g) and malonic acid (2 mmol, 0.21 g) in acetone (60 ml) for 2 h. A clear transparent solution was isolated by filtration and kept in a bottle. Colourless single crystals (1.04 g, yield: 65 %) were obtained after a few days. *M.p.* 419.5 – 420.7 K. Anal. found for C_42_H_40_O_6_Sn_2_: C, 57.38; H, 4.69; Sn, 27.18 %. Calc. for C_42_H_40_O_6_Sn_2_: C, 57.45; H, 4.59; Sn, 27.03 %. FTIR as KBr disc (cm^-1^): ν(COO)_as_ 1656, ν(COO)_s_ 1335, ν(Sn-O) 633. ^1^H-NMR: δ: phenyl photons 7.41-7.48 (18H, m, H*_meta+para_*); 7.65-7.78 (12H, m, H*_ortho_*); CH_2_ 3.59 (2H, s) ppm. ^13^C-NMR: δ: phenyl carbons C*_ipso_* 137.77, C*_ortho_* 136.78, C*_meta_* 128.88, C*_para_* 130.14, CH_2_ 41.65, COO 173.34 ppm.

### Refinement

The water molecule H atoms were located from the difference Fourier map and constrained to ride with the parent atom with *U*_iso_ = 1.5 *U*_eq_(O). All other H atoms were placed in their calculated positions, with C—H = 0.93 – 0.97 Å, and refined using a riding model with *U*_iso_ = 1.2 or 1.5 *U*_eq_(C). A rotating group model was used for the C40 and C42 methyl groups. In the acetone solvent molecule, all atoms were refined isotropically and the C—O and C—C distances were fixed at 1.20 (1) and 1.50 (1) Å, respectively. *EADP* restraints were also imposed on C4:C8 and C37:C39 atom pairs. 4262 Friedel pairs were used in the final refinement to determine the absolute structure. The highest residual electron density peak is located at 1.25 Å from C41 and the deepest hole is located at 0.84 Å from Sn1.

### Crystal data

**Table:**

[Sn_2_(C_6_H_5_)_6_(C_3_H_2_O_4_)(H_2_O)]·C_3_H_6_O	*D*_x_ = 1.512 Mg m^−^^3^
*M**_r_* = 878.12	Mo *K*α radiation, λ = 0.71073 Å
Tetragonal, *I*4	Cell parameters from 9959 reflections
Hall symbol: I -4	θ = 2.4–29.2°
*a* = 23.604 (3) Å	µ = 1.34 mm^−^^1^
*c* = 13.8458 (18) Å	*T* = 100 K
*V* = 7714.2 (17) Å^3^	Plate, colourless
*Z* = 8	0.22 × 0.13 × 0.04 mm
*F*(000) = 3520	

### Data collection

**Table:**

Bruker APEXII DUO CCD area-detector diffractometer	8900 independent reflections
Radiation source: fine-focus sealed tube	7984 reflections with *I* > 2σ(*I*)
graphite	*R*_int_ = 0.099
φ and ω scans	θ_max_ = 27.5°, θ_min_ = 1.7°
Absorption correction: multi-scan (*SADABS*; Bruker, 2009)	*h* = −30→30
*T*_min_ = 0.754, *T*_max_ = 0.953	*k* = −30→30
68157 measured reflections	*l* = −17→17

### Refinement

**Table:**

Refinement on *F*^2^	Secondary atom site location: difference Fourier map
Least-squares matrix: full	Hydrogen site location: inferred from neighbouring sites
*R*[*F*^2^ > 2σ(*F*^2^)] = 0.051	H-atom parameters constrained
*wR*(*F*^2^) = 0.103	*w* = 1/[σ^2^(*F*_o_^2^) + (0.*P*)^2^ + 93.8407*P*] where *P* = (*F*_o_^2^ + 2*F*_c_^2^)/3
*S* = 1.17	(Δ/σ)_max_ = 0.001
8900 reflections	Δρ_max_ = 0.97 e Å^−^^3^
421 parameters	Δρ_min_ = −1.07 e Å^−^^3^
3 restraints	Absolute structure: Flack (1983); 4262 Friedel pairs
Primary atom site location: structure-invariant direct methods	Flack parameter: 0.04 (3)

### Special details

**Table:**

Experimental. The crystal was placed in the cold stream of an Oxford Cryosystems Cobra open-flow nitrogen cryostat (Cosier & Glazer, 1986) operating at 100.0 (1)K.
Geometry. All esds (except the esd in the dihedral angle between two l.s. planes) are estimated using the full covariance matrix. The cell esds are taken into account individually in the estimation of esds in distances, angles and torsion angles; correlations between esds in cell parameters are only used when they are defined by crystal symmetry. An approximate (isotropic) treatment of cell esds is used for estimating esds involving l.s. planes.
Refinement. Refinement of F^2^ against ALL reflections. The weighted R-factor wR and goodness of fit S are based on F^2^, conventional R-factors R are based on F, with F set to zero for negative F^2^. The threshold expression of F^2^ > 2sigma(F^2^) is used only for calculating R-factors(gt) etc. and is not relevant to the choice of reflections for refinement. R-factors based on F^2^ are statistically about twice as large as those based on F, and R- factors based on ALL data will be even larger.

### Fractional atomic coordinates and isotropic or equivalent isotropic displacement parameters (Å^2^)

**Table:**

	*x*	*y*	*z*	*U*_iso_*/*U*_eq_	
Sn1	0.217535 (17)	0.739451 (17)	0.13286 (3)	0.01387 (9)	
Sn2	0.036022 (17)	0.791156 (19)	0.37080 (3)	0.01716 (10)	
O1	0.15759 (19)	0.7609 (2)	0.2618 (3)	0.0145 (9)	
O2	0.12560 (18)	0.8069 (2)	0.3907 (3)	0.0178 (10)	
O3	0.2318 (2)	0.77354 (19)	0.5059 (3)	0.0156 (9)	
O4	0.2431 (2)	0.8673 (2)	0.5045 (3)	0.0192 (10)	
C1	0.1443 (3)	0.7032 (3)	0.0687 (5)	0.0186 (14)	
C2	0.1082 (3)	0.6687 (3)	0.1211 (7)	0.0302 (17)	
H2A	0.1146	0.6622	0.1865	0.036*	
C3	0.0618 (4)	0.6435 (4)	0.0742 (7)	0.044 (2)	
H3A	0.0370	0.6211	0.1099	0.053*	
C4	0.0520 (3)	0.6508 (4)	−0.0206 (6)	0.0311 (12)	
H4A	0.0214	0.6329	−0.0501	0.037*	
C5	0.0877 (4)	0.6850 (4)	−0.0738 (6)	0.043 (2)	
H5A	0.0808	0.6905	−0.1392	0.051*	
C6	0.1346 (3)	0.7117 (4)	−0.0299 (5)	0.0299 (18)	
H6A	0.1587	0.7346	−0.0660	0.036*	
C7	0.2330 (3)	0.8282 (3)	0.1215 (5)	0.0164 (13)	
C8	0.1879 (3)	0.8657 (3)	0.1222 (7)	0.0311 (12)	
H8A	0.1509	0.8523	0.1236	0.037*	
C9	0.1984 (3)	0.9234 (3)	0.1208 (6)	0.0242 (16)	
H9A	0.1682	0.9486	0.1171	0.029*	
C10	0.2528 (3)	0.9440 (3)	0.1246 (7)	0.0337 (18)	
H10A	0.2592	0.9828	0.1258	0.040*	
C11	0.2972 (3)	0.9074 (3)	0.1267 (7)	0.0323 (16)	
H11A	0.3340	0.9212	0.1293	0.039*	
C12	0.2877 (3)	0.8498 (3)	0.1249 (7)	0.0256 (15)	
H12A	0.3183	0.8250	0.1260	0.031*	
C13	0.2702 (3)	0.6933 (3)	0.2302 (5)	0.0165 (13)	
C14	0.3288 (3)	0.6913 (3)	0.2152 (5)	0.0228 (15)	
H14A	0.3449	0.7104	0.1633	0.027*	
C15	0.3635 (3)	0.6601 (4)	0.2795 (6)	0.0330 (19)	
H15A	0.4026	0.6600	0.2713	0.040*	
C16	0.3395 (4)	0.6300 (3)	0.3539 (6)	0.036 (2)	
H16A	0.3623	0.6077	0.3935	0.044*	
C17	0.2809 (3)	0.6324 (3)	0.3708 (6)	0.0316 (17)	
H17A	0.2650	0.6129	0.4225	0.038*	
C18	0.2473 (3)	0.6642 (3)	0.3096 (5)	0.0185 (14)	
H18A	0.2086	0.6663	0.3212	0.022*	
C19	0.0405 (3)	0.7008 (3)	0.3759 (5)	0.0203 (14)	
C20	−0.0015 (4)	0.6672 (4)	0.3374 (6)	0.034 (2)	
H20A	−0.0334	0.6838	0.3097	0.041*	
C21	0.0036 (5)	0.6079 (4)	0.3399 (7)	0.049 (3)	
H21A	−0.0252	0.5855	0.3147	0.059*	
C22	0.0506 (4)	0.5827 (4)	0.3790 (8)	0.043 (2)	
H22A	0.0539	0.5434	0.3802	0.052*	
C23	0.0934 (4)	0.6165 (4)	0.4168 (7)	0.039 (2)	
H23A	0.1255	0.5997	0.4430	0.047*	
C24	0.0887 (3)	0.6751 (3)	0.4158 (5)	0.0251 (16)	
H24A	0.1176	0.6973	0.4415	0.030*	
C25	0.0251 (3)	0.8406 (3)	0.4989 (5)	0.0222 (16)	
C26	−0.0171 (3)	0.8283 (3)	0.5668 (5)	0.0231 (16)	
H26A	−0.0421	0.7986	0.5556	0.028*	
C27	−0.0219 (3)	0.8604 (3)	0.6510 (5)	0.0255 (17)	
H27A	−0.0494	0.8514	0.6966	0.031*	
C28	0.0143 (4)	0.9055 (4)	0.6665 (6)	0.034 (2)	
H28A	0.0108	0.9273	0.7221	0.041*	
C29	0.0555 (3)	0.9182 (4)	0.5996 (6)	0.0315 (19)	
H29A	0.0798	0.9485	0.6103	0.038*	
C30	0.0609 (3)	0.8857 (3)	0.5161 (5)	0.0269 (17)	
H30A	0.0890	0.8945	0.4714	0.032*	
C31	0.0315 (3)	0.8320 (3)	0.2348 (5)	0.0178 (15)	
C32	0.0313 (3)	0.8901 (3)	0.2256 (5)	0.0211 (16)	
H32A	0.0314	0.9121	0.2813	0.025*	
C33	0.0311 (3)	0.9169 (3)	0.1369 (7)	0.0265 (15)	
H33A	0.0307	0.9563	0.1337	0.032*	
C34	0.0316 (3)	0.8851 (4)	0.0528 (6)	0.0266 (18)	
H34A	0.0312	0.9029	−0.0071	0.032*	
C35	0.0327 (3)	0.8264 (4)	0.0589 (5)	0.0253 (17)	
H35A	0.0338	0.8046	0.0030	0.030*	
C36	0.0321 (3)	0.8009 (3)	0.1475 (5)	0.0247 (15)	
H36A	0.0320	0.7615	0.1506	0.030*	
C37	0.1639 (3)	0.7929 (3)	0.3331 (4)	0.0127 (9)	
C38	0.2218 (3)	0.8202 (3)	0.3560 (5)	0.0157 (13)	
H38A	0.2517	0.7985	0.3254	0.019*	
H38B	0.2228	0.8583	0.3301	0.019*	
C39	0.2319 (3)	0.8221 (3)	0.4648 (4)	0.0127 (9)	
O1W	−0.06172 (18)	0.7845 (2)	0.3536 (3)	0.0201 (11)	
H1W1	−0.0802	0.7629	0.3919	0.030*	
H2W1	−0.0759	0.8179	0.3609	0.030*	
O5	0.1396 (14)	0.4466 (12)	0.798 (2)	0.357 (17)*	
C40	0.1640 (7)	0.5374 (7)	0.8785 (16)	0.124 (6)*	
H40A	0.2028	0.5338	0.8586	0.186*	
H40B	0.1616	0.5339	0.9475	0.186*	
H40C	0.1498	0.5738	0.8593	0.186*	
C41	0.1293 (10)	0.4918 (11)	0.8319 (19)	0.179 (11)*	
C42	0.0680 (10)	0.4962 (11)	0.861 (2)	0.214 (12)*	
H42A	0.0490	0.4613	0.8469	0.321*	
H42B	0.0503	0.5266	0.8265	0.321*	
H42C	0.0657	0.5036	0.9294	0.321*	

### Atomic displacement parameters (Å^2^)

**Table:**

	*U*^11^	*U*^22^	*U*^33^	*U*^12^	*U*^13^	*U*^23^
Sn1	0.0163 (2)	0.0163 (2)	0.00904 (16)	0.00189 (17)	0.00150 (19)	−0.00057 (19)
Sn2	0.0135 (2)	0.0274 (2)	0.01057 (17)	0.00137 (17)	0.00065 (19)	−0.0023 (2)
O1	0.017 (2)	0.018 (2)	0.008 (2)	−0.0039 (18)	0.0035 (18)	−0.0030 (17)
O2	0.009 (2)	0.030 (3)	0.015 (2)	0.0023 (19)	0.0021 (17)	−0.0051 (19)
O3	0.022 (3)	0.014 (2)	0.011 (2)	−0.0023 (19)	−0.0067 (18)	0.0026 (18)
O4	0.028 (3)	0.015 (2)	0.016 (2)	0.001 (2)	0.000 (2)	−0.0035 (19)
C1	0.016 (3)	0.021 (4)	0.019 (3)	0.004 (3)	−0.004 (3)	−0.003 (3)
C2	0.024 (4)	0.030 (4)	0.036 (4)	−0.010 (3)	0.000 (4)	−0.004 (4)
C3	0.021 (4)	0.049 (6)	0.061 (6)	−0.015 (4)	0.001 (4)	−0.008 (5)
C4	0.025 (3)	0.031 (3)	0.037 (3)	−0.001 (2)	−0.005 (3)	−0.010 (3)
C5	0.031 (5)	0.065 (7)	0.034 (5)	0.022 (5)	−0.017 (4)	−0.013 (4)
C6	0.027 (4)	0.044 (5)	0.019 (3)	0.009 (4)	−0.003 (3)	−0.009 (3)
C7	0.029 (3)	0.014 (3)	0.006 (3)	−0.003 (3)	−0.003 (3)	0.004 (3)
C8	0.025 (3)	0.031 (3)	0.037 (3)	−0.001 (2)	−0.005 (3)	−0.010 (3)
C9	0.037 (4)	0.016 (3)	0.019 (4)	0.006 (3)	0.000 (3)	0.008 (3)
C10	0.043 (5)	0.017 (3)	0.041 (5)	−0.006 (3)	0.009 (5)	−0.003 (4)
C11	0.037 (4)	0.028 (4)	0.032 (4)	−0.009 (3)	0.001 (4)	0.003 (4)
C12	0.024 (3)	0.017 (3)	0.035 (4)	−0.001 (3)	0.008 (4)	−0.005 (4)
C13	0.021 (3)	0.015 (3)	0.014 (3)	0.003 (3)	−0.003 (3)	−0.002 (3)
C14	0.022 (4)	0.022 (4)	0.024 (4)	0.001 (3)	−0.004 (3)	−0.005 (3)
C15	0.021 (4)	0.043 (5)	0.035 (4)	0.007 (4)	−0.007 (3)	−0.011 (4)
C16	0.050 (5)	0.027 (4)	0.032 (5)	0.012 (4)	−0.019 (4)	−0.012 (4)
C17	0.047 (4)	0.034 (4)	0.014 (3)	0.014 (3)	0.000 (4)	−0.004 (4)
C18	0.019 (4)	0.018 (3)	0.019 (3)	0.003 (3)	0.005 (3)	−0.001 (3)
C19	0.019 (3)	0.035 (4)	0.007 (3)	0.007 (3)	0.003 (3)	−0.006 (3)
C20	0.033 (5)	0.034 (5)	0.036 (4)	0.006 (4)	−0.004 (3)	−0.013 (4)
C21	0.057 (6)	0.032 (5)	0.059 (7)	−0.001 (5)	−0.004 (5)	−0.020 (4)
C22	0.054 (5)	0.031 (4)	0.045 (5)	0.020 (4)	0.010 (5)	−0.004 (5)
C23	0.031 (5)	0.045 (5)	0.041 (5)	0.013 (4)	0.005 (4)	0.006 (4)
C24	0.019 (4)	0.034 (4)	0.022 (4)	0.012 (3)	0.002 (3)	0.009 (3)
C25	0.019 (4)	0.032 (4)	0.016 (3)	0.001 (3)	0.005 (3)	−0.004 (3)
C26	0.015 (3)	0.036 (4)	0.018 (3)	−0.008 (3)	0.004 (3)	0.001 (3)
C27	0.019 (3)	0.043 (5)	0.015 (4)	0.005 (3)	0.005 (3)	−0.003 (3)
C28	0.026 (4)	0.050 (6)	0.026 (4)	0.002 (4)	0.000 (3)	−0.016 (4)
C29	0.025 (4)	0.037 (5)	0.032 (4)	−0.006 (4)	0.006 (3)	−0.017 (3)
C30	0.024 (4)	0.033 (4)	0.024 (4)	−0.003 (3)	0.008 (3)	−0.010 (3)
C31	0.011 (3)	0.030 (4)	0.013 (3)	0.000 (3)	0.005 (3)	0.001 (3)
C32	0.019 (4)	0.029 (4)	0.016 (3)	0.001 (3)	−0.003 (3)	−0.007 (3)
C33	0.025 (4)	0.026 (4)	0.028 (4)	0.003 (3)	0.005 (4)	0.003 (4)
C34	0.023 (4)	0.039 (5)	0.019 (4)	0.003 (4)	−0.001 (3)	0.004 (3)
C35	0.027 (4)	0.036 (5)	0.013 (3)	0.010 (4)	−0.002 (3)	0.001 (3)
C36	0.021 (4)	0.023 (4)	0.030 (4)	−0.001 (3)	0.000 (3)	−0.002 (3)
C37	0.011 (2)	0.020 (2)	0.0064 (18)	0.0028 (18)	−0.0038 (16)	0.0045 (17)
C38	0.019 (3)	0.019 (3)	0.010 (3)	−0.004 (2)	−0.003 (3)	−0.002 (3)
C39	0.011 (2)	0.020 (2)	0.0064 (18)	0.0028 (18)	−0.0038 (16)	0.0045 (17)
O1W	0.009 (2)	0.034 (3)	0.018 (3)	0.0001 (19)	0.0007 (18)	0.002 (2)

### Geometric parameters (Å, °)

**Table:**

Sn1—C1	2.124 (7)	C19—C20	1.378 (11)
Sn1—C7	2.132 (6)	C19—C24	1.403 (9)
Sn1—C13	2.133 (7)	C20—C21	1.405 (12)
Sn1—O3^i^	2.148 (4)	C20—H20A	0.9300
Sn1—O1	2.333 (4)	C21—C22	1.370 (13)
Sn2—C31	2.119 (7)	C21—H21A	0.9300
Sn2—C19	2.137 (7)	C22—C23	1.390 (13)
Sn2—C25	2.139 (7)	C22—H22A	0.9300
Sn2—O2	2.164 (4)	C23—C24	1.387 (12)
Sn2—O1W	2.325 (4)	C23—H23A	0.9300
O1—C37	1.252 (8)	C24—H24A	0.9300
O2—C37	1.249 (7)	C25—C30	1.379 (11)
O3—C39	1.279 (8)	C25—C26	1.400 (10)
O3—Sn1^ii^	2.148 (4)	C26—C27	1.395 (10)
O4—C39	1.230 (8)	C26—H26A	0.9300
C1—C2	1.384 (10)	C27—C28	1.383 (12)
C1—C6	1.399 (10)	C27—H27A	0.9300
C2—C3	1.406 (11)	C28—C29	1.375 (11)
C2—H2A	0.9300	C28—H28A	0.9300
C3—C4	1.343 (13)	C29—C30	1.394 (10)
C3—H3A	0.9300	C29—H29A	0.9300
C4—C5	1.380 (13)	C30—H30A	0.9300
C4—H4A	0.9300	C31—C32	1.376 (11)
C5—C6	1.411 (12)	C31—C36	1.414 (10)
C5—H5A	0.9300	C32—C33	1.382 (11)
C6—H6A	0.9300	C32—H32A	0.9300
C7—C8	1.385 (9)	C33—C34	1.385 (12)
C7—C12	1.387 (9)	C33—H33A	0.9300
C8—C9	1.384 (10)	C34—C35	1.387 (12)
C8—H8A	0.9300	C34—H34A	0.9300
C9—C10	1.373 (10)	C35—C36	1.368 (10)
C9—H9A	0.9300	C35—H35A	0.9300
C10—C11	1.358 (11)	C36—H36A	0.9300
C10—H10A	0.9300	C37—C38	1.544 (9)
C11—C12	1.379 (9)	C38—C39	1.525 (8)
C11—H11A	0.9300	C38—H38A	0.9700
C12—H12A	0.9300	C38—H38B	0.9700
C13—C14	1.401 (10)	O1W—H1W1	0.8551
C13—C18	1.403 (9)	O1W—H2W1	0.8618
C14—C15	1.416 (11)	O5—C41	1.189 (10)
C14—H14A	0.9300	C40—C41	1.498 (10)
C15—C16	1.375 (12)	C40—H40A	0.9600
C15—H15A	0.9300	C40—H40B	0.9600
C16—C17	1.403 (12)	C40—H40C	0.9600
C16—H16A	0.9300	C41—C42	1.507 (10)
C17—C18	1.383 (10)	C42—H42A	0.9600
C17—H17A	0.9300	C42—H42B	0.9600
C18—H18A	0.9300	C42—H42C	0.9600
			
Sn1···O1	2.333 (4)	Sn2···O2	2.164 (4)
Sn1···O3^i^	2.148 (4)	Sn2···O1W	2.325 (4)
Sn1···C1	2.124 (7)	Sn2···C19	2.137 (7)
Sn1···C7	2.132 (6)	Sn2···C25	2.139 (7)
Sn1···C13	2.133 (7)	Sn2···C31	2.119 (7)
			
C1—Sn1—C7	120.3 (3)	C19—C20—H20A	120.0
C1—Sn1—C13	122.2 (3)	C21—C20—H20A	120.0
C7—Sn1—C13	116.7 (3)	C22—C21—C20	120.8 (9)
C1—Sn1—O3^i^	93.1 (2)	C22—C21—H21A	119.6
C7—Sn1—O3^i^	89.1 (2)	C20—C21—H21A	119.6
C13—Sn1—O3^i^	96.9 (2)	C21—C22—C23	119.2 (8)
C1—Sn1—O1	85.0 (2)	C21—C22—H22A	120.4
C7—Sn1—O1	87.0 (2)	C23—C22—H22A	120.4
C13—Sn1—O1	88.9 (2)	C24—C23—C22	120.8 (8)
O3^i^—Sn1—O1	174.03 (17)	C24—C23—H23A	119.6
C31—Sn2—C19	119.1 (3)	C22—C23—H23A	119.6
C31—Sn2—C25	118.9 (3)	C23—C24—C19	119.9 (8)
C19—Sn2—C25	121.5 (3)	C23—C24—H24A	120.0
C31—Sn2—O2	94.9 (2)	C19—C24—H24A	120.0
C19—Sn2—O2	96.8 (2)	C30—C25—C26	118.7 (7)
C25—Sn2—O2	85.3 (2)	C30—C25—Sn2	119.4 (5)
C31—Sn2—O1W	83.6 (2)	C26—C25—Sn2	121.9 (6)
C19—Sn2—O1W	89.2 (2)	C27—C26—C25	120.5 (7)
C25—Sn2—O1W	90.1 (2)	C27—C26—H26A	119.8
O2—Sn2—O1W	173.79 (18)	C25—C26—H26A	119.8
C37—O1—Sn1	131.5 (4)	C28—C27—C26	119.8 (7)
C37—O2—Sn2	125.4 (4)	C28—C27—H27A	120.1
C39—O3—Sn1^ii^	119.4 (4)	C26—C27—H27A	120.1
C2—C1—C6	119.7 (7)	C29—C28—C27	120.0 (7)
C2—C1—Sn1	121.3 (5)	C29—C28—H28A	120.0
C6—C1—Sn1	118.9 (6)	C27—C28—H28A	120.0
C1—C2—C3	119.1 (8)	C28—C29—C30	120.3 (8)
C1—C2—H2A	120.4	C28—C29—H29A	119.9
C3—C2—H2A	120.4	C30—C29—H29A	119.9
C4—C3—C2	122.1 (9)	C25—C30—C29	120.7 (7)
C4—C3—H3A	119.0	C25—C30—H30A	119.6
C2—C3—H3A	119.0	C29—C30—H30A	119.6
C3—C4—C5	119.4 (8)	C32—C31—C36	116.1 (7)
C3—C4—H4A	120.3	C32—C31—Sn2	122.4 (5)
C5—C4—H4A	120.3	C36—C31—Sn2	121.5 (6)
C4—C5—C6	120.7 (8)	C31—C32—C33	122.6 (7)
C4—C5—H5A	119.7	C31—C32—H32A	118.7
C6—C5—H5A	119.7	C33—C32—H32A	118.7
C1—C6—C5	119.0 (8)	C32—C33—C34	119.9 (7)
C1—C6—H6A	120.5	C32—C33—H33A	120.1
C5—C6—H6A	120.5	C34—C33—H33A	120.1
C8—C7—C12	118.6 (6)	C33—C34—C35	119.4 (7)
C8—C7—Sn1	119.8 (5)	C33—C34—H34A	120.3
C12—C7—Sn1	121.2 (5)	C35—C34—H34A	120.3
C9—C8—C7	119.5 (7)	C36—C35—C34	119.6 (7)
C9—C8—H8A	120.3	C36—C35—H35A	120.2
C7—C8—H8A	120.3	C34—C35—H35A	120.2
C10—C9—C8	120.9 (7)	C35—C36—C31	122.5 (7)
C10—C9—H9A	119.5	C35—C36—H36A	118.8
C8—C9—H9A	119.5	C31—C36—H36A	118.8
C11—C10—C9	119.8 (7)	O2—C37—O1	125.3 (6)
C11—C10—H10A	120.1	O2—C37—C38	113.5 (5)
C9—C10—H10A	120.1	O1—C37—C38	121.2 (6)
C10—C11—C12	120.1 (7)	C39—C38—C37	110.7 (5)
C10—C11—H11A	120.0	C39—C38—H38A	109.5
C12—C11—H11A	120.0	C37—C38—H38A	109.5
C11—C12—C7	120.9 (7)	C39—C38—H38B	109.5
C11—C12—H12A	119.5	C37—C38—H38B	109.5
C7—C12—H12A	119.5	H38A—C38—H38B	108.1
C14—C13—C18	118.7 (6)	O4—C39—O3	125.3 (6)
C14—C13—Sn1	119.9 (5)	O4—C39—C38	120.1 (6)
C18—C13—Sn1	121.4 (5)	O3—C39—C38	114.4 (6)
C13—C14—C15	119.7 (7)	Sn2—O1W—H1W1	118.8
C13—C14—H14A	120.2	Sn2—O1W—H2W1	108.1
C15—C14—H14A	120.2	H1W1—O1W—H2W1	105.9
C16—C15—C14	120.1 (8)	C41—C40—H40A	109.5
C16—C15—H15A	119.9	C41—C40—H40B	109.5
C14—C15—H15A	119.9	H40A—C40—H40B	109.5
C15—C16—C17	120.7 (8)	C41—C40—H40C	109.5
C15—C16—H16A	119.7	H40A—C40—H40C	109.5
C17—C16—H16A	119.7	H40B—C40—H40C	109.5
C18—C17—C16	119.0 (8)	O5—C41—C40	135 (3)
C18—C17—H17A	120.5	O5—C41—C42	111 (3)
C16—C17—H17A	120.5	C40—C41—C42	111 (2)
C17—C18—C13	121.7 (7)	C41—C42—H42A	109.5
C17—C18—H18A	119.2	C41—C42—H42B	109.5
C13—C18—H18A	119.2	H42A—C42—H42B	109.5
C20—C19—C24	119.2 (7)	C41—C42—H42C	109.5
C20—C19—Sn2	121.7 (6)	H42A—C42—H42C	109.5
C24—C19—Sn2	119.0 (5)	H42B—C42—H42C	109.5
C19—C20—C21	120.1 (8)		
			
C1—Sn1—O1—C37	−166.9 (6)	O2—Sn2—C19—C20	−159.2 (6)
C7—Sn1—O1—C37	−46.0 (6)	O1W—Sn2—C19—C20	22.5 (6)
C13—Sn1—O1—C37	70.7 (6)	C31—Sn2—C19—C24	117.4 (6)
C31—Sn2—O2—C37	−55.5 (6)	C25—Sn2—C19—C24	−70.7 (6)
C19—Sn2—O2—C37	64.6 (5)	O2—Sn2—C19—C24	17.9 (6)
C25—Sn2—O2—C37	−174.2 (6)	O1W—Sn2—C19—C24	−160.4 (6)
C7—Sn1—C1—C2	−128.6 (6)	C24—C19—C20—C21	1.2 (12)
C13—Sn1—C1—C2	40.5 (7)	Sn2—C19—C20—C21	178.3 (7)
O3^i^—Sn1—C1—C2	140.6 (6)	C19—C20—C21—C22	−1.0 (14)
O1—Sn1—C1—C2	−45.0 (6)	C20—C21—C22—C23	0.2 (15)
C7—Sn1—C1—C6	55.4 (6)	C21—C22—C23—C24	0.4 (14)
C13—Sn1—C1—C6	−135.5 (6)	C22—C23—C24—C19	−0.3 (12)
O3^i^—Sn1—C1—C6	−35.4 (6)	C20—C19—C24—C23	−0.6 (11)
O1—Sn1—C1—C6	138.9 (6)	Sn2—C19—C24—C23	−177.8 (6)
C6—C1—C2—C3	−1.0 (11)	C31—Sn2—C25—C30	−58.1 (7)
Sn1—C1—C2—C3	−177.0 (6)	C19—Sn2—C25—C30	130.0 (6)
C1—C2—C3—C4	1.6 (14)	O2—Sn2—C25—C30	34.9 (6)
C2—C3—C4—C5	−1.4 (14)	O1W—Sn2—C25—C30	−140.9 (6)
C3—C4—C5—C6	0.7 (13)	C31—Sn2—C25—C26	122.5 (6)
C2—C1—C6—C5	0.4 (11)	C19—Sn2—C25—C26	−49.4 (7)
Sn1—C1—C6—C5	176.4 (6)	O2—Sn2—C25—C26	−144.5 (7)
C4—C5—C6—C1	−0.2 (12)	O1W—Sn2—C25—C26	39.7 (6)
C1—Sn1—C7—C8	34.8 (7)	C30—C25—C26—C27	−1.4 (12)
C13—Sn1—C7—C8	−134.9 (6)	Sn2—C25—C26—C27	178.0 (6)
O3^i^—Sn1—C7—C8	127.8 (6)	C25—C26—C27—C28	1.7 (12)
O1—Sn1—C7—C8	−47.7 (6)	C26—C27—C28—C29	−1.0 (13)
C1—Sn1—C7—C12	−152.9 (6)	C27—C28—C29—C30	0.1 (13)
C13—Sn1—C7—C12	37.4 (7)	C26—C25—C30—C29	0.5 (12)
O3^i^—Sn1—C7—C12	−59.9 (6)	Sn2—C25—C30—C29	−178.9 (6)
O1—Sn1—C7—C12	124.6 (6)	C28—C29—C30—C25	0.2 (13)
C12—C7—C8—C9	3.6 (12)	C19—Sn2—C31—C32	−176.7 (6)
Sn1—C7—C8—C9	176.1 (6)	C25—Sn2—C31—C32	11.2 (7)
C7—C8—C9—C10	−4.0 (13)	O2—Sn2—C31—C32	−76.1 (6)
C8—C9—C10—C11	2.2 (14)	O1W—Sn2—C31—C32	97.8 (7)
C9—C10—C11—C12	0.0 (15)	C19—Sn2—C31—C36	−0.8 (7)
C10—C11—C12—C7	−0.3 (15)	C25—Sn2—C31—C36	−172.9 (6)
C8—C7—C12—C11	−1.5 (13)	O2—Sn2—C31—C36	99.8 (6)
Sn1—C7—C12—C11	−173.9 (7)	O1W—Sn2—C31—C36	−86.3 (6)
C1—Sn1—C13—C14	129.2 (5)	C36—C31—C32—C33	0.7 (11)
C7—Sn1—C13—C14	−61.3 (6)	Sn2—C31—C32—C33	176.8 (6)
O3^i^—Sn1—C13—C14	31.2 (6)	C31—C32—C33—C34	−0.6 (11)
O1—Sn1—C13—C14	−147.3 (5)	C32—C33—C34—C35	−0.4 (11)
C1—Sn1—C13—C18	−50.4 (6)	C33—C34—C35—C36	1.3 (12)
C7—Sn1—C13—C18	119.0 (6)	C34—C35—C36—C31	−1.3 (12)
O3^i^—Sn1—C13—C18	−148.5 (5)	C32—C31—C36—C35	0.2 (11)
O1—Sn1—C13—C18	33.0 (5)	Sn2—C31—C36—C35	−175.9 (6)
C18—C13—C14—C15	0.3 (10)	Sn2—O2—C37—O1	−10.4 (9)
Sn1—C13—C14—C15	−179.3 (5)	Sn2—O2—C37—C38	168.2 (4)
C13—C14—C15—C16	2.6 (11)	Sn1—O1—C37—O2	169.6 (4)
C14—C15—C16—C17	−3.8 (12)	Sn1—O1—C37—C38	−8.9 (9)
C15—C16—C17—C18	2.0 (11)	O2—C37—C38—C39	39.1 (8)
C16—C17—C18—C13	1.0 (11)	O1—C37—C38—C39	−142.2 (6)
C14—C13—C18—C17	−2.1 (10)	Sn1^ii^—O3—C39—O4	−24.6 (9)
Sn1—C13—C18—C17	177.5 (5)	Sn1^ii^—O3—C39—C38	150.1 (4)
C31—Sn2—C19—C20	−59.7 (7)	C37—C38—C39—O4	−125.3 (7)
C25—Sn2—C19—C20	112.2 (7)	C37—C38—C39—O3	59.7 (7)

Symmetry codes: (i) −*x*+1/2, −*y*+3/2, *z*−1/2; (ii) −*x*+1/2, −*y*+3/2, *z*+1/2.

### Hydrogen-bond geometry (Å, °)

**Table:**

Cg1 and Cg2 are the centroids of the C31–C36 and C7–C12 benzene rings, respectively.

**Table:**

*D*—H···*A*	*D*—H	H···*A*	*D*···*A*	*D*—H···*A*
O1W—H1W1···O4^iii^	0.86	1.90	2.663 (6)	148
C5—H5A···O5^iv^	0.93	2.59	3.38 (3)	144
C26—H26A···O4^iii^	0.93	2.50	3.356 (8)	154
C8—H8A···Cg1	0.93	2.83	3.701 (8)	157
C17—H17A···Cg2^ii^	0.93	2.79	3.571 (9)	142
C38—H38B···Cg2	0.97	2.97	3.613 (8)	125

Symmetry codes: (iii) *y*−1, −*x*+1, −*z*+1; (iv) −*y*+1/2, *x*+1/2, −*z*+1/2;  (ii) −*x*+1/2, −*y*+3/2, *z*+1/2.
